# Pulmonary Gangrene

**Published:** 1850-12

**Authors:** 


*Pulmonary Gangrene.* By MORETON STILLE, M.D., of Philadelphia.

In the thirteenth volume of the Prague Quarterly Journal of Practical Medicine, may be found an article upon Pulmonary Gangrene, by Dr. Fischel, physician to the Insane Asylum of the city of Prague. It is one of those valuable contributions to medical literature, which, from being in a language not generally understood, is not available to the mass of English readers. As, however, it relates to a subject rarely submitted to extended investigation, and contains many interesting observations, we propose to make use of it as a basis for the remarks we have to offer on this subject, and compare the opinions of the author with those of other writers. The disease treated of, although a rare one, is at the same time of much pathological interest, and on account of its infrequency, is not as well understood as it ought to be. Reports of the nature of that prepared by Dr. Fischel deserve to be made generally known, for it is by such laborious investigations that the bounds of our science are enlarged, and a wider field of profitable study reclaimed.

In the space of six years, viz., from 1839 to 1845 inclusive, there were made, at the Prague institution for Morbid Anatomy 3437 autopsies. Of this number, 3102 came from the General Hospital, the Lying-in and Foundling Hospitals, and 335 from the Insane Asylum. The cases of pulmonary gangrene among the former were 55, among the latter were 25, which is a proportion among the sane of 1.6, and among the insane of 7.4 to every 100 autopsies. This excess of cases among the insane was constant in each year, a fact which seems to preclude the idea, that the great mortality from gangrene of the lung was due to an epidemic influence. The form of insanity was melancholy in 12, epilepsy in 5, mania in 4, and in 4 idiocy.

Dr. Fischel considers, that this predominance of the cases among the insane, shows a greater liability to the disease upon their part ; an opinion which is in harmony with the views of Genest and Guislain. He thinks also that its more frequent occurrence among those suffering under the depressing forms of mental disease, confirms the idea of its being dependent upon an impairment of the nervous energy, but does not venture to locate this supposed lesion, as some have done, in the pneumogastric nerves. Its exciting cause in the majority of his cases was insufficient and improper food. Others enumerated were loss of blood, abuse of ardent spirits, great physical exertion, and prolonged venereal excess. This statement, gives, of course, but a very imperfect view of the etiology of gangrene of the lung. The persons who came under Dr. Fischel's observations were insane, and confined in a public charitable institution, their energies were exhausted by poverty, excess and actual disease, as well as by prolonged confinement, and voluntary abstinence from food. It is not not difficult, therefore, to perceive how all these causes may have concurred to produce a condition of the system favoring the development of gangrene. It would not be correct, however, to regard them as the ordinary causes of the disease as it occurs in persons not under the same unfavorable hygienic circumstances. If its incidental development in the course of diseases of a typhous character be excepted, it will be found that it usually occurs under the influence of exposure to cold and dampness, during periods of depression following great excitement either of a mental or bodily nature. It cannot, indeed, be denied, that there is a certain kind of constitution in which it seems to be more readily developed then in others, but it is very difficult to describe it. Schonlein says, that it attacks principally young people of delicate skin and florid complexion who have given themselves up to an intemperate course of living. Canstatt collected twenty-two fatal cases, in sixteen of which the constitutional strength of the patient was noted, and twelve of them are described as being remarkably healthy persons. *Med. Clinik. Bd.* 3.) Dr. Gerhard says that " gangrene occurs in exhausted subjects, either affected with diseases calculated to weaken the powers of the system, or enfeebled by a life of intemperance, but there are exceptions to this rule. (*Am. Journ.*, vol. xviii, p. 301.) It is probable, that these statements, which appear to be somewhat at variance with each other, (and more of the same kind might easily be cited,) result from the frequent want of correspondence between the apparent muscular strength and the constitutional power of resistance to disease. While the first remains but little impaired, the foundations of the latter may have been long secretly undermined; cases exemplifying this truth are of daily observation, especially in our large hospitals, both in the medical and surgical practice ; it being no uncommon event, that men of large stature and powerful frame, but of intemperate habits, succumb rapidly under injuries or diseases, apparently of a trifling character. Gangrene occurs in such persons most readily, being at one time the consequence and at another the cause of phlebitis, or the index of a general or of a local deficient vitality.

Dr. Fischel makes the usual division of pulmonary gangrene into two kinds, viz., the circumscribed and the diffused. Although Cruveilhier *Path. Anat.* t. i. liv. ii.) thinks that both varieties are equally common, we believe he is the only writer of any authority who expresses such an opinion. Lawrence out of sixty-eight cases met with diffused gangrene only six times, and Laennec only twice in twenty-four years. In the Report for the year 1848 of the Vienna Institute of Morbid Anatomy, by Dr. Lauthner, we find that 1069 post mortem examinations were made in the year, and of this large number there were only five cases of gangrene of the lung, one only of which was of the diffused kind. In Dr. Fischel's eighty cases there were but four diffused. Dr. Fischel's results confirm also the general impression that the disease occurs preferably in the lower portion of the lung, and chiefly in that of the right side; except, indeed, where it is consecutive to tubercular disease, when it may occur, of course, in any part of the organ so affected.

We have not met with any description of the forming stage of pulmonary gangrene, drawn from actual inspection of the lung, with the exception of a case reported by Dr. Gerhard, (loc. sit. Case VIII.) In this case, which was one of diffused gangrene, the death of the patient took place at a very early period in the disease; the texture of the lung was not broken down, although infiltrated with serum, diminished in consistence, and of a gangrenous odor. The later stages of the gangrenous process are well known, and may be easily recognised, but, in the absence of any very positive knowledge of its mode of commencement, it is usual and convenient to ascribe it to inflammation. It is very certain, however, that not being an ordinary result of inflammation, it must depend upon something extrinsic to this process, but not essential to it. If inflammation does not really include (as it is sometimes made to do) every possible morbid process, it is far more simple to confine it within the limits of stasis and exudation, and to regard the phenoma of resorption or of organization of the effused fibrin as processes of reparation, complimentary to, but not parts of the inflammation. There really exists no such thing as too violent inflammation in this sense; it is by its effusion that it becomes excessive or dangerous, since it cannot, in the ascending scale, go beyond the limit of an effusion of a more or less plastic material. When the blood vessels have been relieved by its discharge, the phenomena which follow are those of repair, and this process is more or less complete, according to the tissue affected, and the constitutional strength of the individual. The sentiment cannot be too often repeated, paradoxical as it may seem, that inflammation is, *essentially*, a conservative process, and that when, by interference with the functions of organs essential to life, it becomes lethal, it is so, either by its extent, or by the feeble recuperative power of the patient. If gangrene supervene upon inflammation, it is therefore to be attributed, not to the violence of this, but to causes which, although difficult to appreciate, are known to act by impairing or destroying the constitutional power of resistance. The engorgement and hepatization which are found around the gangrened portion of the lung, cannot be regarded as manifesting a prior stage of the process of mortification, but are rather an indication of an effort made by nature, to throw up a barrier against the extension of the disease, by which the dead tissue is circumscribed, and the source of the contamination isolated from the rest of the organ. It is the same process, essentially, which is seen in gangrene of the extremities, the progress of which it is designed to limit; when it does not occur, it is plain that no reliance can be placed upon the recuperative powers of the system. We may seem to be begging the question if we assert that gangrene is never a result of pneumonia, but as the direct anatomical proof of the fact cannot be procured, it is fair to this as a matter of general experience, that so common a disease as is pulmonary inflammation, equally rare is gangrene of the lung, and that if it be a consequence of violent pneumonia, the fact is a very remarkable one that it should occur so seldom. Indeed, all authors who have given particular attention to this point, agree that it does never succeed to a frank pneumonia; which is certainly equivalent to an admission, that if it depends at all upon inflammation, it is assuredly not due to an excess of it. Dr Hodgkin (*Lectures—Mucous Membrane*) says, that it "generally, if not always, seems to require some peculiarity in the constitution of the individual, rather than merely to depend on intense inflammation;" also, "it sometimes affects small spots scattered through the substance of the lungs; which quickly lose their vitality, without the precurrence of the ordinary symptoms of inflammation of the lungs." Genest and Grisolle do not think that inflammation alone can produce it. The latter has never seen it follow a well characterized pneumonia. (*Trait prat de la Pneumonie*, p. 355.) Dr. Gerhard (*loc. cit.*) says, "In every instance in which the patients were seen in the early stages of the disease, I could distinguish the humid rhonchi, indicating bronchitis, or at least the secretion of liquid into the bronchial tubes. There was, in no instance, bronchial respiration, dull sound on percussion, or other unequivocal evidence of pneumonia; and in one case only was there even reason to suspect that pneumonia may have preceded gangrene." Laennec (*Dis. of Chest*, p. 207) says, that "it can scarcely be ranked among the terminations of pulmonary inflammation, and still less can it be considered as the consequence of its intensity." The opinion of some eminent pathologists, particularly of Carswell, Piorry, Cruveheilier, and Schrœdervan der Kolk, that the obliteration or obturation of the arteries of the lung, is the proximate cause of pulmonary gangrene, is hardly tenable, and that for reasons analogous to those we have offered above. This obturation, it is reasonable to suppose, is the consequence and not the cause of the death of the pulmonary structure, and depends possibly upon an inflammation of the minute pulmonary vessels, in consequence of the reception of the corrosive products of the gangrened portion of the lung, as well as upon the effusion of plastic lymph in the structure around them, by which they are compressed and their canals effaced. Dr. Fischel justly observes, that this inflamed state of the pulmonary vessels, has never been found as a primary affection. But however easy it may be to assign valid reasons against these views, it is by no means equally so, to say what is the proximate cause.

Dr. Fischel, indeed, in the spirit of the German "*Krasenlehre*,"—or doctrine of crases,—feels satisfied with referring it to a hypinosis, or to a deficiency of fibrine in the blood; but this is not a sufficient explanation for one very plain reason, viz., that this condition of the blood exists in several other pathological states of the system, in which, nevertheless, no gangrene occurs, or is dreaded. The view advocated by Genest, and Dr. Law, of Dublin, that it is a consequence of pulmonary apoplexy, seems to have much in its favor; for it is not difficult to conceive, that the effused blood, if it remain in the lung, may become putrefied by the action of the air, and become the source of the disease in that part with which it is in immediate contact. We believe that gangrene may originate in this manner, but there are many cases which cannot be explained by it. Hæmorrhage from the lungs is not unfrequently one of the first signs observed, yet it is more common in the progress of the disease than at its beginning,—a result of its progress, not the force which sets it in motion. The opinion of Dr. Stokes, always of great value, and particularly so in relation to this disease, is that the accidental putrefaction of blood effused in the lungs, cannot be reckoned even as an ordinary cause of pulmonary gangrene." He has not seen any cases of the change from one of these diseases to the other; and is of the opinion that where a pulmonary clot does become putrid, "the change is in itself a proof of gangrenous disposition pre-existing."—*Dub. Journal*, Feb., 1850.

While it is of practical importance that the idea of the dependency of gangrene of the lung upon inflammation should be combated, and its relation to pulmonary apoplexy considered as exceptional, it is not necessary for a correct knowledge of its treatment that an acquaintance with the exact nature of the process should first be possessed. For, knowing that all those causes which depress the nervous energy and vitiate the blood, may take part in its production, and that it appears, at times, to originate in consequence of the deprivation of sufficient nutriment, a rational treatment can be founded thereupon. In Dr. Fischel's cases, this last cause played an important part, and a large and interesting portion of his paper is taken up with a narrative of the means by which he sought to remedy it. We do not propose to enumerate these, nor the remedies recommended by various writers, as we would thereby trespass too much upon the space allotted to us. We have desired to call attention merely in a general way, to the anatomical characters of this formidable disease, and would take the liberty of referring the reader, for a minute account of the same, to a translation of Rokitansky's description in Copland's Medical Dictionary. In order to understand the interesting process by which, in favorable cases, a cure is accomplished, we might bring together the results of the observations of Dr. Fischel, and the accurate clinical reports of Dr. Gerhard, before referred to. By such a comparison it will be seen how beneficial is the result of the inflammation surrounding the gangrened portion of the lungs; the extension of the disease being limited by an exudation of plastic lymph around the dead tissue. This exuded fibrin forms a wall of various thickness around around the cavity left by the separation of the slough, and after the latter has been removed by expectoration, the walls of the cavity, in course of time, gradually contract and approach each other, and sometimes finally unite, forming a dense cicatrix in the spot formerly occupied by the gangrenous eschar.—*Medical Examiner*.

